# Phantom Hernia: A Rare Sequela of Herpes Zoster Infection

**DOI:** 10.7759/cureus.63095

**Published:** 2024-06-25

**Authors:** Aditya Sharma, Ritika Khanna, Seema Khanna, Rahul Khanna

**Affiliations:** 1 Department of General Surgery, Institute of Medical Sciences, Banaras Hindu University, Varanasi, IND; 2 Department of Dermatology & Venereology, Institute of Medical Sciences, Banaras Hindu University, Varanasi, IND

**Keywords:** neurological complication, pseudohernia, herpes zoster, anterior abdominal wall, phantom hernia

## Abstract

The herpes zoster (HZ) virus first manifests as varicella, or chickenpox, in children and remains dormant in the dorsal root ganglia of the nerves. The vesicular eruptions that might be painful develop along a dermatome when the virus is reactivated. While postherpetic neuralgia is a well-known side effect of herpes simplex, there are well-reported motor consequences as well. Segmental zoster paresis is an uncommon motor consequence of herpes zoster that resembles an anterior abdominal wall hernia but does not require surgery in contrast to an actual abdominal wall hernia.

We present a similar case of a 46-year-old male who presented with classic herpes zoster rash and phantom hernia as a rare sequela of this condition.

## Introduction

Herpes zoster is a dermatological disorder with rare neurological complications and often presents with grouped vesicles in the affected dermatome. It is caused by the varicella-zoster virus (VZV), which is activated in the dorsal root ganglia [1]. The most frequent symptom of herpes zoster virus infection is sensory abnormalities, but motor neuropathy is an uncommon side consequence [2]. Abdominal paresis is a rare motor consequence of segmental zoster, with an incidence of about 0.7% [1,2]. Between 1% and 5% of people suffer from motor weaknesses; the most common condition is Ramsay-Hunt syndrome. The complications associated with this condition include pseudo-obstruction, paralytic ileus, bladder dysfunction, hemidiaphragm paralysis, visceral neuropathy, and abdominal pseudohernia. Most occurrences of segmental zoster paresis have been documented in middle-aged and older individuals, typically affecting the face and limbs [3]. The weakening of the abdominal wall, resulting in flank or abdominal bulges that resemble hernias, is relatively rarely reported in Case Studies [1,3].

Herein, we are reporting a case of a 46-year-old male who initially developed herpes zoster infection of the flanks and later developed a weakness of the anterior abdominal wall (phantom hernia) as a rare sequela of the disease.

## Case presentation

**Figure 1:**
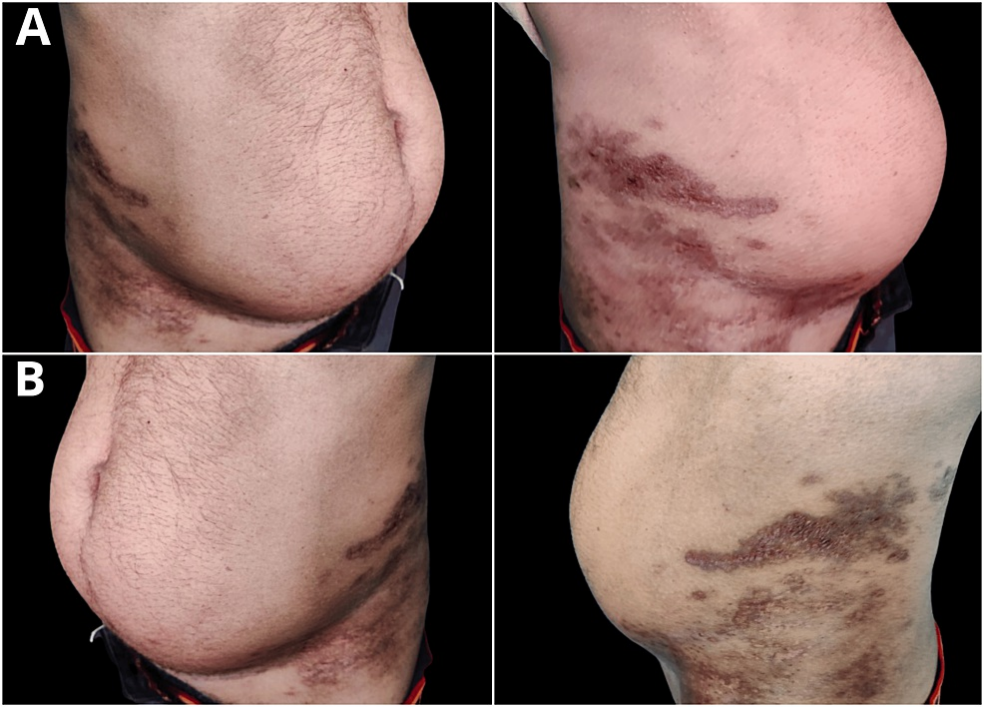
A clinical picture showing a healed herpetic skin rash with dimensions of 16x12 and 18x14 cm and extension in the right (A) and left (B) flanks, respectively

A 46-year-old gentleman attended our outpatient clinic, complaining mostly of a rash that had gradually progressed from its insidious beginning to bilateral flanks, back, and abdomen. The abdominal wall weakness and hence protrusion were observed concurrently, and they were not associated with any other complaints of pain or fever. He was known to be diabetic for the past two years, for which he was taking oral hypoglycemic agents. On examination, a healed herpetic skin rash with dimensions of 16x12 and 18x14 cm, extending from the regions of the back to the bilateral flanks and abdomen, was noted in the right and left flanks, respectively. Both limb activity and muscle strength were normal. There was a noticeable bulging of the abdomen on both sides, which became more noticeable and apparent when the abdominal pressure was elevated, as shown in Figure 1.

After a dermatological opinion, an abdominal ultrasonogram was done to delineate the abdominal wall defect in order to differentiate it from a true hernia. There was no history of any surgical intervention in the region involved. It was found that the patient did not have the abdominal wall defect or mass that was first assumed. Rather, the patient suffered from segmental herpes zoster, a motor consequence of the disease that presents as pseudohernia due to abdominal wall weakness. It was recommended that the patient make more follow-up visits and take oral acyclovir 800 mg five times a day for seven days, pregabalin 75 mg once a day for a total of 14 days, and methylcobalamin 1500 mcg fast release for 14 days.

## Discussion

It is thought that the herpes zoster virus infects the spinal cord at the anterior horn level after the virus spreads from the dorsal root ganglia to the brain, although the exact underlying mechanism is unknown [4]. Although sensory involvement manifesting as radiating nerve root pain is the common manifestation of this viral infection, motor nerve root involvement has also been reported in the literature. The pathological findings can be explained by the electrophysiological consequences of the disease, leading to ganglion lesions, severe neuritis, and degeneration of the motor and sensory roots. 

For instance, the clear-cut diagnosis of segmental zoster abdominal paresis is supported by the easy testing of the paraspinal muscles with electromyography, despite the fact that they are often implicated and challenging to assess clinically [4,5]. Although the clinical and radiological symptoms of this medical condition are described by the phrase "phantom hernia or zoster pseudohernia," it fails to address the segmental nature of the disease [6]. The involvement of the nerve root can be assessed by gadolinium-diethylenetriaminepentaacetic acid (DTPA)-enhanced nuclear magnetic resonance imaging [5,6].

We believe segmental zoster abdominal paresis/paralysis is the most appropriate term to use. Reactivation of the latent varicella-zoster virus can frequently impact other muscles in the same spinal segment, even though this may not always be clinically apparent. It is important to recognize a pseudo-hernia because, unlike an incisional hernia, it is not associated with the risks of obstruction, incarceration, or strangulation. Also, surgical correction is not required in a pseudo-hernia.

## Conclusions

Herpes simplex and an abdominal wall bulging is a diagnostic combination, as seen in the present case of pseudo-hernia. Because the rash could develop after the swelling, close observation might be beneficial. Dermatologists and surgeons, particularly, need to be sensitized about the occurrence of these events in order to treat patients effectively.

## References

[1] Healy C, McGreal G, Lenehan B, McDermott EW, Murphy JJ (1998). Self-limiting abdominal wall herniation and constipation following herpes zoster infection. QJM.

[2] Broadbent WH (1866). Case of herpetic eruption in the course of branches of the brachial plexus, followed by partial paralysis in corresponding motor nerves. Br Med J.

[3] Sharma A, Singh SK, Srivastava V, Pratap A, Ansari MA (2024). Segmental abdominal paresis attributed to herpes zoster infection mimicking an abdominal hernia: an interesting case from a surgical unit of a tertiary healthcare center. Cureus.

[4] Chernev I, Dado D (2013). Segmental zoster abdominal paresis (zoster pseudohernia): a review of the literature. PM R.

[5] Yoo J, Koo T, Park E, Jo M, Kim MS, Jue MS (2019). Abdominal pseudohernia caused by herpes zoster: 3 case reports and a review of the literature. JAAD Case Rep.

[6] Giuliani A, Galati G, Parisi L (2006). Postherpetic paresis mimicking an abdominal herniation. Acta Derm Venereol.

